# Effects of Larval Diet on the Male Reproductive Traits in the West Indian Sweet Potato Weevils *Euscepes postfasciatus* (Coleoptera: Curculionidae)

**DOI:** 10.3390/insects13040389

**Published:** 2022-04-14

**Authors:** Chihiro Himuro, Kinjo Misa, Atsushi Honma, Yusuke Ikegawa, Tsuyoshi Ohishi, Norikuni Kumano

**Affiliations:** 1 Okinawa Prefectural Plant Protection Center, Naha 902-0072, Japan; kinjomis@pref.okinawa.lg.jp (K.M.); honma.tetrix@gmail.com (A.H.); y.ikegawa224@gmail.com (Y.I.); ooishits@pref.okinawa.lg.jp (T.O.); 2 Ryukyu Sankei Co., Ltd., Naha 902-0072, Japan; 3 Faculty of Agriculture, University of Ryukyus, Nishihara 903-0213, Japan; 4 Laboratory of Insect Ecology, Department of Life Science and Agriculture, Obihiro University of Agriculture and Veterinary Medicine, Obihiro 080-8555, Japan; nrkumano@gmail.com

**Keywords:** ejaculate, accessory gland, seminal fluid protein, refractory period, SDS-PAGE, mass-rearing, sweet potato

## Abstract

**Simple Summary:**

In insects, it is known that the diet during the larval stage affects traits in the adult stage. However, it is still unclear how it affects reproductive traits such as ejaculation. The ejaculate contains many proteins and therefore requires much nutrition, so the larval diet strongly influences it. Males of the West Indian sweet potato weevil *Euscepes postfasciatus* use accessory gland substances to inhibit remating by females. Crossing experiments were conducted using lines reared on artificial diets or sweet potato tubers during the larval stage, and the refractory period was examined. The results showed that the larval stage diet had a significant effect on the refractory period of females. We also found one protein of approximately 15 kDa in size expressed only in the treatments reared on sweet potatoes. To our knowledge, this is the first study to show that larval diet qualitatively influences male ejaculate and female refractory period.

**Abstract:**

Larval diet significantly affects adult traits, although less is known about how they affect reproductive traits. Males of West Indian sweet potato weevil *Euscepes postfasciatus* deliver a remating inhibitor along with sperm to their mates during mating, leading to a refractory period (the period before females mate again). Crossing experiments were conducted using lines reared on artificial diets, including sweet potato powder (AD) or sweet potato tubers (SP) during the larval stage, and the refractory period was examined. We also examined whether the larval diet qualitatively or quantitatively altered male ejaculate. The results showed that the refractory period was significantly longer in the SP treatment than in the AD treatment for males and females. There was no significant difference in ejaculate volume. However, the number of sperm in the testes-seminal vesicles complex was significantly higher in the SP treatment. Additionally, SDS-PAGE revealed that the ejaculate was qualitatively different depending on the larval diet, and one protein of approximately 15 kDa in size was expressed only in the SP treatments. Revealing how larval diet affects reproductive traits in adult males will help shed light on the diverse evolution of insect mating systems and reproductive behavior.

## 1. Introduction

It is well known that qualitative and quantitative differences in diet can affect genetically identical traits in organisms. The larval diet significantly influences larval and adult traits in insects. In the cotton bollworm *Helicoverpa armigera* (Hübner), the body color of the last instar larvae was strongly influenced by the part of the plant on which they fed [1]. Studies on the black soldier fly *Hermetia illucens* (Linnaeus) showed that qualitative differences in larval diet affected the physiological and morphological development, including larval and pupal periods, mortality rate, body size, and ovary size [2]. The diet during the developmental period has a significant physiological and morphological effect on life history and reproductive traits in the adult stage. Reproductive traits especially, have a significant effect on fitness of the sexes. In exploring the evolution of reproductive traits, it is necessary to clarify how diet during the larval stage affects them. One of the most important reproductive traits in males is ejaculates, which are composed of sperm and seminal fluid proteins and peptides (Sfps) produced mainly in the accessory glands of the male reproductive tract, transferred from males to females during mating. There are more than 200 seminal fluid molecules in *Drosophila melanogaster* [3,4,5,6,7], several hundred in mice [8], between 50 and 100 in honeybees [9], and surprisingly, more than 900 in humans [10], more than 1000 in mosquitoes [11,12]. These substances are involved in various events related to female and male reproduction, such as sperm survival and sperm competition, acceleration of female egg-laying and female lifespan, and inhibition of female remating (reviewed [13,14,15]). The production of these ejaculates requires nutrients. The quantity and quality of diet in the larval and adult stages are expected to significantly impact the male reproductive traits.

The effects of diet during the ‘adult stage’ on reproductive traits and behaviors have been widely studied in both sexes. Males of *Drosophila grimshawi* (Oldenberg) fed nutritionally rich diets have been reported to court more vigorously and mate more successfully than males fed less nutritionally poor diets [16]. In the Mediterranean fruit fly *Ceratitis capitata* (Wiedemann), males fed a protein-poor diet also copulated less often than males fed a protein-rich diet, probably due to a less frequent courtship activity, but they have a greater probability of sperm transfer and storage [17,18]. While, males fed a protein-rich diet have a greater ability to inhibit female receptivity [18,19,20]. In the Queensland fruit fly *Bactrocera tryoni* (Froggatt), a protein diet (yeast hydrolysate) in the adult stage increased the probability of sperm storage and the amount of sperm stored and reduced the probability of female remating [21]. Additionally, protein-fed males of the West Indian fruit fly *Anastrepha obliqua* (Macquart) exhibited longer copulation duration and induced longer refractory periods in their mates than protein-deprived males [22,23].

Additionally, the effects of ‘larval’ diet on reproductive traits also have been studied in both sexes. Experimentally reducing the amount of diet or nutrients in a larval diet significantly reduced the number and the length of sperm produced in adult stage in the Indian meal moth *Plodia interpunctella* (Hübner) [24] and the fruit fly *Drosophila melanogaster* (Meigen) [25]. Studies were also conducted on females to examine how the larval diet affects the size of the ovaries and stage of development of oocytes (e.g., [2,26,27]). Nutritional deficiency in larvae decreases sperm production [24], male remating behavior, fecundity of their mates [28], and adult traits related to reproductive success [29]. Some studies reported that competition for resources during the larval stage might affect ejaculates during the adult stage [30,31]. Within tephritids, *C. capitata* larvae that were reared on a protein-rich medium had more nutrient reserves (e.g., increased lipids) as adults, and sexual maturation was faster than in adults derived from a protein-deprived diet [32]. The reproductive behavior and traits are greatly influenced by larval diet. Some research on the effects of larval diet on male ejaculate has been examined for effects on sperm quality and quantity, but less is known about the effects on Sfps from an accessory gland.

The West Indian sweet potato weevil *Euscepes postfasciatus* (Fairmaire) (Coleoptera: Curculionidae) is a major pest of sweet potato, *Ipomoea batatas* (L.) Lam. (Convolvulaceae) in the South Pacific, the Caribbean Basin, parts of Central and South America [33,34], and the southern islands of Japan [35,36]. In Okinawa Prefecture, Japan, where the weevils are infested, a sterile insect technique (SIT) program to eradicate this weevil is underway [37]. SIT programs release large numbers of sterile insects over long periods. Therefore, it is essential to establish an economic mass-rearing system (Knipling [38], reviewed [39]). At Okinawa Prefectural Plant Protection Center (OPPPC), Naha, Japan, weevils are mass-produced using artificial diets, and many improvements have been made on the composition of artificial diets and the amount needed to improve the yield (e.g., [40,41,42,43,44]). The mating behavior of *E. postfasciatus* has been reported (e.g., [45,46]). The mated females temporarily became unavailable for additional mating [46,47,48], because males inhibit female remating for 14 days on average through Sfps from an accessory gland [47]. It was shown that males have a pair of testis-seminal vesicle complexes and three types of accessory glands, of which a substance derived from accessory gland B acts to inhibit female re-mating [47]. The effect seems to depend on the amount of ejaculate (N. Kumano, personal communication, 25 March 2021). It has been suggested that inducing this change in mated females is of benefit to males by decreasing the likelihood of sperm competition. In *D. melanogaster*, it was known that the ejaculate changes depending on the rearing environment (density) during the larval stage [49], but it is not fully known how other rearing environments, such as larval diet quality, affect the ejaculate.

This study examined the effect of the larval diet on the refractory period of *E. postfasciatus* by crossing within and between weevil groups reared with an artificial diet containing sweet potato powder or sweet potato tuber in their larval stage. We also compared the male reproductive traits between the groups: mating duration, the amount of sperm in the testes-seminal vesicles complex, the amount of ejaculate, the effect of accessory gland substances on inhibiting female remating. In addition, each ejaculate was quantitatively analyzed and compared using SDS-PAGE. Lastly, the amount of sweet potato powder in the artificial diets was varied to examine its effect on male reproductive traits. Mass-rearing insect quality related to reproductive traits could greatly contribute to increasing the efficiency of SIT. One objective of this study is to increase the efficiency of SIT by improving the diet during the larval stage in *E. postfasciatus*.

## 2. Materials and Methods

### 2.1. Experimental Insects

Weevils were obtained from mass-reared stock populations established from 138,487 wild-caught adult weevils collected in Itoman City, Okinawa Island (26°7′ N, 127°42′ E), Japan, in November and December 2004 [37,50]. They were successively reared using fresh sweet potato root tubers for 35 generations and about 230 generations using an artificial diet [40,43,51,52] at 25 ± 1 °C with a photoperiod of L14:D10 (lights were on from 04:00–18:00) at OPPPC. Contents of the artificial diet for larvae of *E. postfasciatus* were reported by Ohishi et al. [40] (see Table S1). At OPPPC, two diets are used for the mass production of this weevil: artificial diet (hereafter abbreviated as AD) for successive generations and sweet potato tubers (SP) for the insects to be released as part of the SIT program [51]. The detailed rearing methods using AD and SP have been described by Ohishi et al. [51] and Okinawa Prefectural Plant Protection Center [52]. Eight weeks after weevils’ egg suspension inoculation, the AD was disassembled to collect virgin adults that had just emerged (AD treatment). For the SP treatment, we collected 100 sexually mature individuals from the AD treatment and placed them in a plastic container with a mesh lid (2.3 L, 205 × 140 × 80 mm) with ca. 180 g SP as diet and substance for oviposition. After a week, all weevils were removed, and the inoculated SP was placed in the container for eight weeks. The SP was checked every two or three days to collect virgin adults that had just emerged (SP treatment). The average yield per 100 g of diet was 157 individuals in the AD treatment and 124.3 individuals in the SP treatment. Newly emerged adults from AD and SP were sexed by observing the external characteristics of the genitalia under a stereomicroscope (Nikon SMZ645, Nikon Corporation, Tokyo, Japan) [53]. The center of the elytron of each male was marked with a ca. 1 mm diameter spot of white paint (uni POSCA PC-5M; Mitsubishi Pencil Co., Ltd., Tokyo, Japan). Individuals of the same sex and age for each treatment (AD and SP) were housed together for at least 14 days with no more than 25 individuals per plastic jar (0.2 L, top: 95 mm diameter, bottom: 75 mm diameter, 45 mm height). In both AD and SP treatments, adults were fed sweet potato tubers (ca. 80 g; the cut surface of the sweet potato tubers was coated with paraffin wax to prevent decay). The adults were reared at 25 ± 2 °C, with a photoperiod of L14:D10 (lights on from 00:00 to 14:00 h). In all experiments, we used sexually mature virgin individuals 14–21 d after emergence (DAE).

### 2.2. Experiment 1. Effects of the Larval Diet of Males and Females on the Refractory Period of Females

We measured the length of the refractory period (the recovery phase after mating, i.e., the time between one mating and the next) of females in the experimental setting as a behavioral assay. Crossing experiments were performed using male and female AD and SP treatments. Four treatment groups were established as follows: male from SP treatment with female from SP treatment, *n* = 43; male from SP treatment with female from AD treatment, *n* = 35; male from AD treatment with female from SP treatment, *n* = 45; male from AD treatment with female from AD treatment, *n* = 37. A pair from each of the four treatment groups were placed into small plastic Petri dishes (Falcon Easy Grip Petri Dish, 35 mm in diameter, 10 mm in height; Becton Dickinson, Franklin Lakes, NJ, USA), and their mating behavior was observed for 3 h (14:00–17:00 h) at 25 ± 2 °C under dark conditions using a red LED flashlight (DOL2D2J; Energizer, St. Louis, MO, USA) and timer (CBM HS01, Tokyo, Japan). This weevil species is active at night [54]; therefore, we conducted our observations under dark conditions. The sequence of the mating behavior of *E. postfasciatus* is described below [46,48,55]. When the male found the female, he climbed on the female’s back (pre-copulatory mate guarding) and stimulated the elytra with his middle and hind legs. The male then moved slightly backward, placed his abdomen under the female, and inserted his genitals. At that time, the male and female bodies were at right angles, and the male ejaculated. After completing copulation, the male climbed onto the female’s back again (postcopulatory mate guarding). Their mating behavior lasted about 40 min [45,46], and most of the sperm were transferred to the female storage organ between 3 and 5 min after the initiation of copulation [45]. We recorded the start and finish times of copulation to calculate mating duration. The collected females were individually reared in plastic containers (top: 65 mm in diameter, bottom: 45 mm in diameter, 40 mm in depth) containing sweet potato root (ca. 20 g) as a diet and an oviposition substrate. Sweet potato roots were changed every 10 d. Every day after mating, the females underwent mating experiments with virgin males to measure their refractory period. The experiment was conducted in the same setting as the first mating. However, male counterparts for each day were randomly chosen from a 14–21 DAE group to avoid possible confounding effects (e.g., male quality and age). These observation procedures were continued until the focal females exhibited remating behavior or died.

### 2.3. Experiment 2. Effects of the Larval Diet on Male Reproductive Traits

We obtained 14–21 DAE, sexually mature virgin individuals from AD and SP treatments. As an indicator of body size, the left elytron length was measured using imaging software (WraySpect, Wraymer Inc., Osaka, Japan) under a stereomicroscope (YS05T NobitaT, Micronet Inc., Saitama, Japan). We then investigated the effects of larval diet on male reproductive traits using the methods described below.

#### 2.3.1. Number of Sperm in the Testes-Seminal Vesicles Complex

To examine the difference in the number of sperm in the testes and seminal vesicles, males were dissected in saline (Otsuka normal saline, 4.5 g of NaCl per 0.5 L of distilled water, pH 6.4; Otsuka Pharmaceutical Factory, Inc., Tokushima, Japan) under a binocular microscope (MZ-6; Leica Microsystems GmbH, Wetzlar, Germany). Internal organs of the abdomen, including the reproductive tract with the accessory glands, seminal vesicles, testes, and ejaculatory duct [47], and digestive organs were removed using forceps (SP treatment, *n* = 30; AD treatment, *n* = 29). The seminal vesicle is attached to the center of the testis and we were unable to separate the seminal vesicle from the testis by manipulation with a stereomicroscope. Therefore, to assess the progress of sperm production, we compared the number of sperm in the testes and seminal vesicles (hereafter referred to as testes-seminal vesicles complex). The removed testes-seminal vesicles complexes were placed in Eppendorf tubes (0.5 mL) containing 100 µL saline. Each extract was homogenized with forceps and a toothpick for 30 s. Using a micropipette, 10 µL of the solution was placed in one spot on a five-window speculum plate (UR157S; Sekisui Chemical Co., Ltd., Tokyo, Japan). Using an eyepiece micrometer with squares and an optical microscope (Nikon Eclipse 50i; Nikon Corporation, Tokyo, Japan), the number of sperm within 0.25 mm^2^ (0.5 mm by 0.5 mm square) is measured at ten randomly selected points per window. Since one-tenth of the sample (i.e., 10/100 µL) were investigated in a 2.5 mm^2^ (10 points × 0.25 mm^2^) area of each window (120 mm^2^: 10 mm × 12 mm), we can estimate the total sperm number by multiplying the counts by 480.

#### 2.3.2. Number of Transferred Sperm into the Spermatheca

We examined the difference in the number of transferred sperm into the spermatheca. The mating experiments were carried out under the same conditions and methods as in Experiment 1 described above. Pairs of SP and AD treatments were placed in a small plastic Petri dish and allowed to mate for 4 h starting at 13:30. After mating, the mated females were captured and stored in a refrigerator at 4 °C (SP treatment, *n* = 10; AD treatment, *n* = 12). They were dissected in saline under a binocular microscope using forceps, and the spermatheca was removed the next day (see Figure S1, [47]). The removed spermatheca was crushed with sharp forceps in distilled water (10 μL), dropped on a glass slide, mixed to homogenize the spermatheca suspension in the water, and covered with a cover glass (18 mm × 18 mm). Under an optical microscope fitted with an eyepiece micrometer, the number of sperm within 0.5 mm × 0.5 mm were counted randomly at 20 locations within the cover glass. The number of sperm in the spermatheca, i.e., the number of transferred sperm into the spermatheca, was calculated as the ratio of the cover glass area (324 mm^2^) to the number of sperm in 5 mm^2^ (20 points × 0.25 mm^2^).

#### 2.3.3. Seminal Fluid Substances

To examine how seminal fluid substances differ between males of SP and AD treatments, we conducted SDS-PAGE.

##### SDS-PAGE

Reproductive tracts, including accessory glands, testes-seminal vesicles complex, and ejaculatory ducts of virgin males (15-DAE sexually mature), were dissected in Milli-Q water under a binocular microscope (MZ-6; Leica Microsystems GmbH, Wetzlar, Germany), removed, and stored in 1.5 mL Eppendorf tubes containing Milli-Q water (60 μL) chilled in ice. We collected 60 pairs of accessory glands B (AGB), C (AGC), and testes-seminal vesicles complex (TSVC) from each treatment (Figure S1, [47]). Each sample was then frozen at −60 °C prior to protein extraction. The frozen samples were thawed and homogenized using a disposable homogenizer (BioMasher II; NIPPI Inc., Tokyo, Japan) in 120 μL of buffer (contents: 2 mL of 0.5 M Tris-HCl (pH 6.8), 4 mL of 10% SDS, 1.2 mL of *β*-mercaptoethanol, 2 mL of glycerol, a drop of 1% bromophenol blue (BPB), and 0.8 mL distilled water per 10 mL of buffer). Samples were heated at 95 °C for 5 min and then centrifuged at 15,000 rpm for 10 min at 4 °C. The supernatant containing soluble proteins was subjected to SDS-PAGE.

SDS-polyacrylamide gel electrophoresis was carried out using a WSE-1150 PageRun Ace (ATTO, Tokyo, Japan) at 21 mA for 80 min. at room temperature (25 ± 2 °C). The samples were loaded on 12.5% SDS–PAGE gels (e-PAGEL, E-R15L, ATTO) in running buffer (EzRun, ATTO; 25 mM Tris, 192 mM Glycine, 0.1% (*w*/*v*) SDS). The EzProtein Ladder (WSE-7020, ATTO, Tokyo, Japan) was used as a molecular weight marker. The volume of each sample was 10 μL, and the molecular weight marker was 5 μL. The gels were stained with Coomassie brilliant blue (CBB) (EzStain Aqua AE-1340, ATTO) for 3 h and washed with distilled water. Gels from the study were collected, digitalized, and analyzed using ImageJ (ver. 1.53a, National Institute of Health, Bethesda, MD, USA, http://imagej.nih.gov/ij/, accessed on 9 July 2021. The following equation was derived from the size of the marker and its distance from the top of the gel to estimate the size of the protein.

(1) $$Y=294786x^{-1.371}$$

where *Y* is the distance from the gel top to the target protein measured using ImageJ, and *x* is the estimated protein size.

### 2.4. Experiment 3. Effects of the Amount of Sweet Potato Powder in the Artificial Diet on the Refractory Period of Females

To investigate the effects of the larval diet during the refractory period in females, four treatments were prepared by changing the amount of sweet potato powder in the AD to 0, 10, 25, and 50 g/L (female from AD treatment, 0 g/L, *n* = 23; 10 g/L, *n* = 16; 25 g/L, *n* = 28; 50 g/L, *n* = 26, male from AD treatment, 0 g/L, *n* = 30; 10 g/L, *n* = 28; 25 g/L, *n* = 27; 50 g/L, *n* = 26). The weevils were reared in the same way described for the AD treatment. Weevils were obtained from each treatment, and mating experiments to investigate the refractory period were carried out using the same procedure as in Experiment 1. The mating partners were fixed to those collected from the SP treatment.

### 2.5. Experiment 4. Effects of the Amount of Sweet Potato Powder in the Artificial Diet on Reproductive Traits

Weevils were obtained for each treatment (see Exp. 3) and the number of sperm in the testes-seminal vesicles complexes (0 g/L *n* =6; 10 g/L *n* = 7; 25 g/L *n* = 7; 50 g/L *n* = 8) and the number of transferred sperm into the spermatheca (0 g/L *n* =6; 10 g/L *n* = 5; 25 g/L *n* = 11; 50 g/L; *n* = 11) were measured in the same way as in Experiment 2. In the experiment measuring the number of transferred sperm into the spermatheca, females from the SP treatment were used in all treatments to exclude the influence of females.

### 2.6. Statistics

The effects of diet on male and female larval stage on the refractory period of females were analyzed using the Weibull model (parametric survival analysis), only one which is both relative event rates and relative extension in survival time (in this case, whether the females have remated or not) can be simultaneously estimated [56,57]. The factors (i.e., male and female larval diet) included in the model were analyzed using the Wald test. In cases where the female died without remating, the lifetime period after the first mating was recorded as censored data. The effects of the amount of sweet potato powder in AD on male and female larval stages on the refractory period of the female were analyzed using the same methods. We used the GLM with a Gaussian distribution and identity link function to analyze the effect of diet on male and female larval stage on the mating duration, the effects of larval diet and male body size on the number of sperm in testes-seminal vesicles complex, the effect of larval diet on the number of transferred sperm into the spermatheca, the effects of the amount of sweet potato powder in AD on the number of sperm in the testes-seminal vesicles complex, the number of transferred sperms into the spermatheca. The significance level for all the tests was set at 5%. All analyses were conducted using JMP software (version 14.3 [58], Cary, NC, USA).

## 3. Results

### 3.1. Experiment 1. Effects of the Larval Diet of Males and Females on the Refractory Period of Females

**Figure 1:**
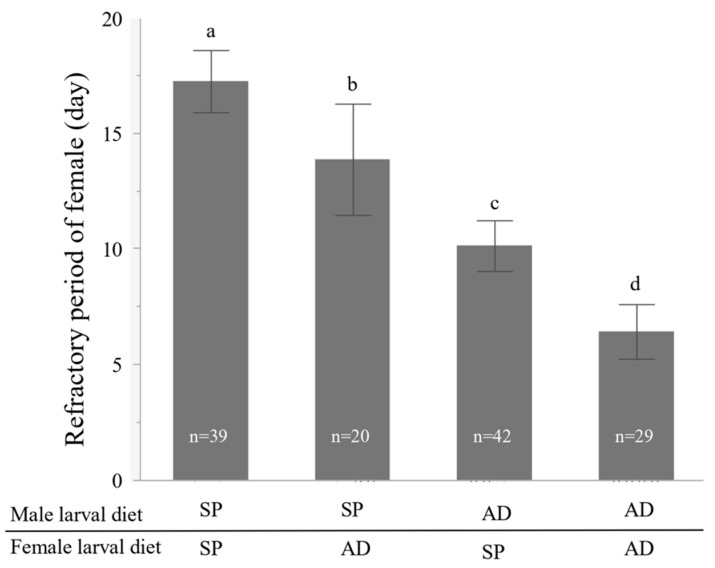
Effects of the male and female larval diet on the refractory period of females (days; mean ± S.E.). On the y-axis, the upper represents the diet of the male larval stage and the lower the diet of the female larval stage. There are two types of larval diet, sweet potato (SP) and artificial diet (AD). Different letters show significant differences (Wald test, *p* < 0.05).

**Figure 2:**
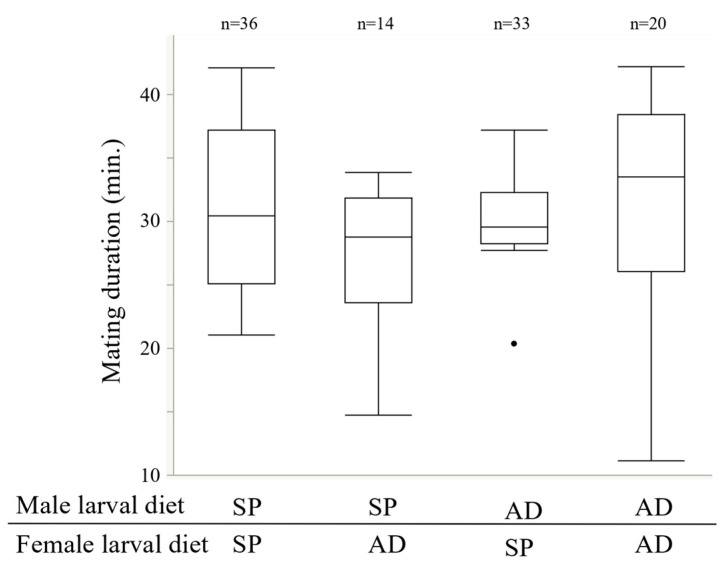
Effects of the male and female larval diet on the mating duration (min.). Boxplots represent medians (thick lines), the first and third quartile ranges (box perimeters), minimum and maximum (whiskers), and outliers (small circles). The explanation of the Y-axis is the same as in Figure 1.

Both male and female larval diets had a significant effect on the length of the refractory period (Figure 1, Weibull model, χ^2^ = 26.51, *p* < 0.0001; Wald test, male larval diet; estimated value = 0.30, χ^2^ = 22.81, *p* < 0.0001; female larval diet estimated value = 0.13, χ^2^ = 4.60, *p* < 0.05; male larval diet × female larval diet estimated value = −0.06, χ^2^ = 0.83, *p* = 0.36). The larval diet of females and males did not significantly affect the mating duration (Figure 2, GLM, male larval diet L-R (likelihood ratio) χ^2^ = 2.01, *p* = 0.17; female larval diet L-R χ^2^ = 1.22, *p* = 0.27; male larval diet × female larval diet L-R χ^2^ = 3.23, *p* = 0.07).

### 3.2. Experiment 2. Effects of the Larval Diet on Male Reproductive Traits

**Figure 3:**
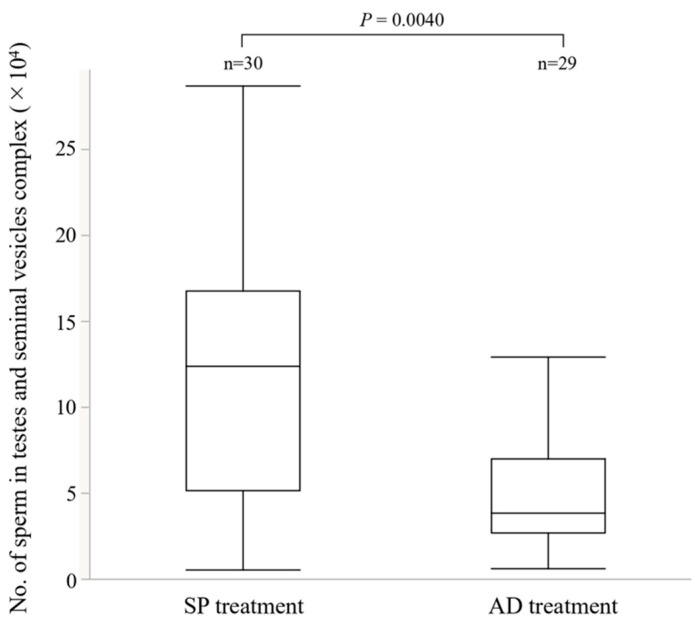
Comparison of the number of sperm in the testes-seminal vesicles complex of different male treatments. Boxplots represent medians (thick lines), the first and third quartile ranges (box perimeters), minimum and maximum (whiskers). The diet of the larvae significantly affected the number of sperm in the testes -seminal vesicles complex (GLM, larval diet; χ^2^ = 8.2629, *p* = 0.0040).

**Figure 4:**
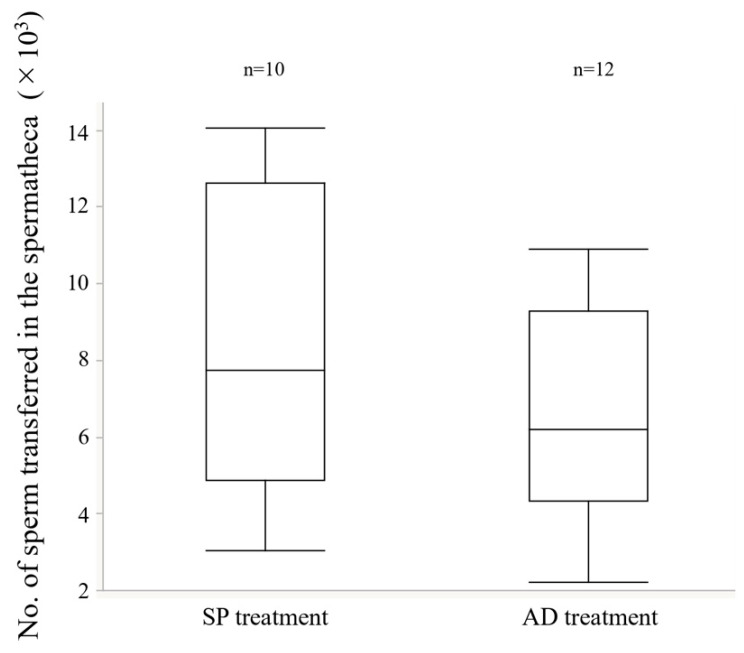
Comparison of the number of sperm transferred into the spermatheca to different treatments. Boxplots represent medians (thick lines), the first and third quartile ranges (box perimeters), and minimum and maximum (whiskers).

**Figure 5:**
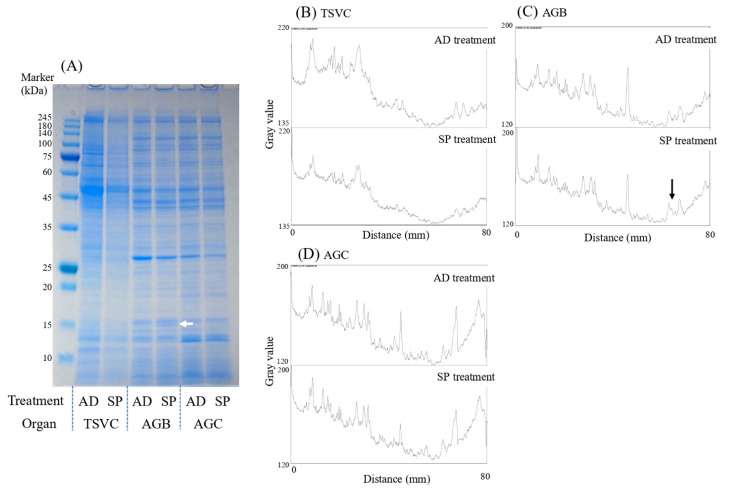
(**A**) a representative gel image of the male testes-seminal vesicles complex (TSVC) and accessory gland B (AGB) and C (AGC) from the artificial diet (AD) and sweet potato (SP) treatments of E. postfasciatus. The white arrow shows a male SP treatment-specific protein of about 15 kDa in size. Comparisons of gray values between AD and SP treatments in testes-seminal vesicles complex (**B**), AGB (**C**), AGC (**D**). The black arrow in (**C**) shows a SP treatment-specific protein of about 15 kDa in size.

The diet of the larvae significantly affected the number of sperm in the testes-seminal vesicles complex, however, male body size had no effect (Figure 3, GLM, larval diet L-R χ^2^ = 8.26, *p* < 0.01; male body size L-R χ^2^ = 0.52, *p* = 0.47; larval diet × male body size; L-R χ^2^ = 0.02, *p* = 0.89). In contrast, the number of transferred sperm into the spermatheca was not affected by the diet of the male larval stage (Figure 4, GLM, L-R χ^2^ = 1.35, *p* = 0.24). Anatomically, there was no difference between AD and SP males (Figure S1, [47]). SDS-PAGE results are shown in Figure 5A. The TSVC of both AD and SP males contained more than 19 different proteins with no qualitative differences (Figure 5A,B and Table S2). In AGB, more than 26 common proteins and one SP-specific protein of approximately 15 kDa in size were found (Figure 5A,C and Table S2). More than 27 different proteins without qualitative differences were found in AGC (Figure 5A,D and Table S2).

### 3.3. Experiment 3. Effects of the Amount of Sweet Potato Powder in the Artificial Diet on the Refractory Period of Females

**Figure 6:**
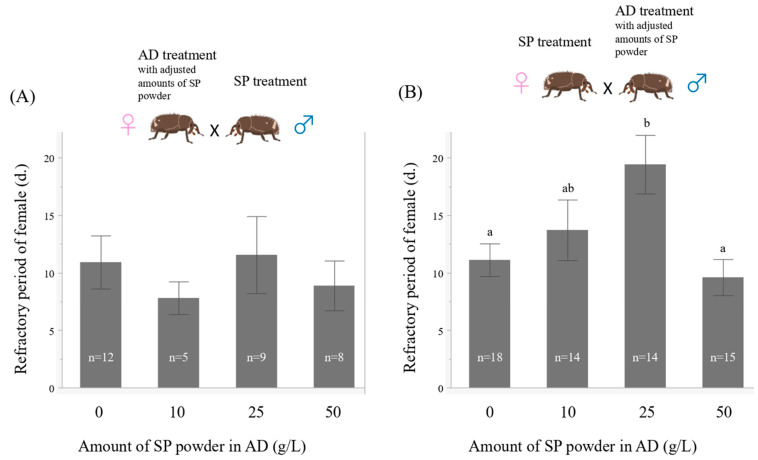
Relationship between the amount of sweet potato powder and the length of the refractory period of females (days; mean ± S.E.). (**A**) refractory period of females, obtained from AD treatment with adjusted amounts of SP powder, mated with males of SP treatment. (**B**) refractory period of females of SP treatment mated with males obtained from AD treatment with adjusted amounts of SP powder. Different letters show significant differences (*p* < 0.05).

In AD females, the length of the refractory period was not significantly influenced by the amount of sweet potato powder (Figure 6A, Weibull model, χ^2^ = 2.02, *p* = 0.57; Wald test, the amount of sweet potato powder χ^2^ = 2.31, *p* = 0.51). In contrast, in AD males, the amount of sweet potato powder in the larval stage significantly influenced the length of the refractory period of females (Figure 6B, Weibull model, χ^2^ = 11.13, *p* < 0.05; Wald test, the amount of sweet potato powder χ^2^ = 12.14, *p* < 0.01). The length of the refractory period of females mated with the 25 g/L treated males was significantly longer than females mated with the 0 g/L and 50 g/L treated males.

### 3.4. Experiment 4. Effects of the Amount of Sweet Potato Powder in the Artificial Diet on Reproductive Traits

**Figure 7:**
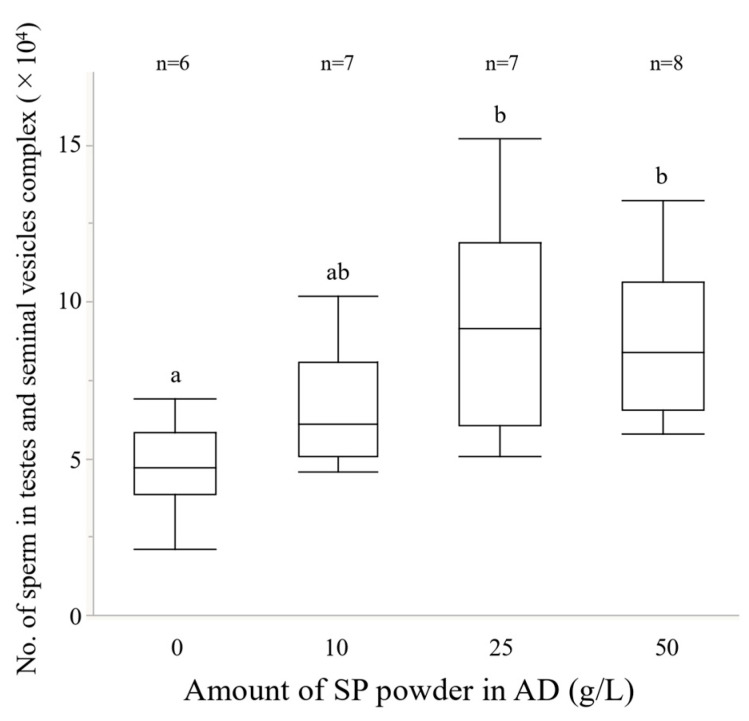
Relationship between the amount of sweet potato powder and the number of sperm in testes-seminal vesicles complexes. Boxplots represent medians (thick lines), the first and third quartile ranges (box perimeters), minimum and maximum (whiskers). The diet of the larvae significantly affected the number of sperm in the testes-seminal vesicles complex (GLM; L-R χ^2^ = 11.81, *p* < 0.01). Different letters show significant differences (*p* < 0.05).

**Figure 8:**
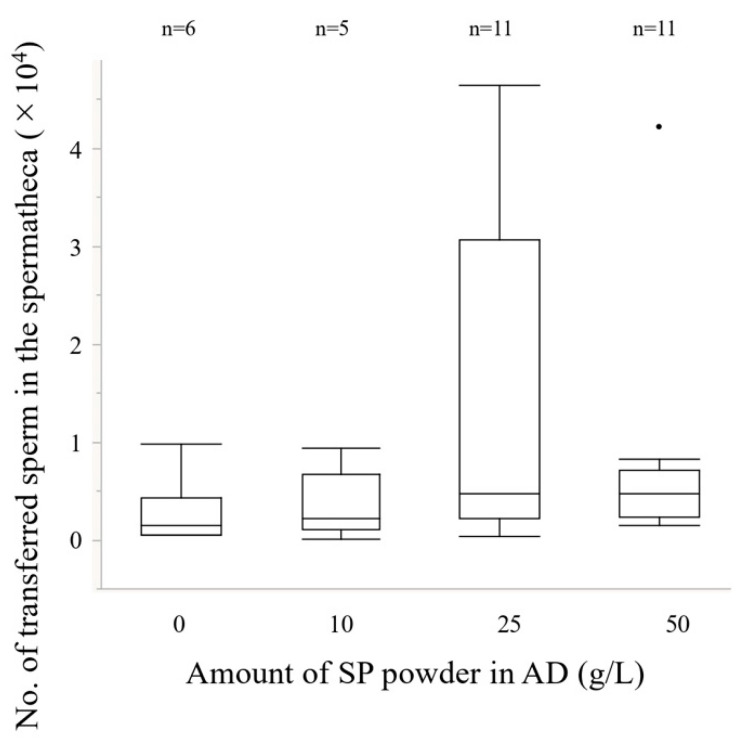
Relationship between the amount of SP (sweet potato) powder and the number of transferred sperm into the spermatheca. Boxplots represent medians (thick lines), the first and third quartile ranges (box perimeters), minimum and maximum (whiskers), and outliers (small circles). The larval diet did not significantly affect the number of transferred sperm into the spermatheca (GLM; L-R χ^2^ = 3.92, *p* = 0.27).

The amount of sweet potato powder in the AD significantly affected the number of sperm in the testes-seminal vesicles complex (Figure 7, GLM L-R χ^2^ = 11.81, *p* < 0.01). The refractory period was significantly shorter in the 0 g/L treatment of males than in the 10 g/L and 50 g/L treatments (GLM with the sequential Bonferroni method, *p* < 0.05). In contrast, the amount of sweet potato powder in the AD group did not significantly affect the number of transferred sperm into the spermatheca (Figure 8, GLM; L-R χ^2^ = 3.92, *p* = 0.27).

## 4. Discussion

### 4.1. Effects of Larval Diet on the Female Refractory Period

Our experiments showed that the larval diet of both males and females affected the refractory period of females in *E. postfasciatus* (Figure 1). The refractory period of females that mated with males from the AD treatment was shorter than that of females mating with males from the SP treatment. In addition, the refractory period of females reared on AD was shorter than those reared on SP. Although several studies reported that qualitative and quantitative differences in the adult diet affect female remating in fruit flies, for example, male Mediterranean fruit flies, *Ceratitis capitata*, and Queensland fruit flies, *Bactrocera tryoni* fed high-quality diets are more likely to inhibit female remating than low-quality fed males [18,22], to our knowledge, this is the first report to show that larval diet affects female remating. Since both sexes were affected by the larval diet, different mechanisms in males and females influence the length of the refractory period in females of this weevil.

### 4.2. Effects of Larval Diet on the Reproductive Traits

Many mechanisms are involved in the refractory periods of females. Two are related to male seminal fluids, mainly composed of sperm and Sfps produced in the accessory glands of the male reproductive tract (Reviewed [13,14,15]). One effect associated with this physiological change is triggered by the amount of sperm stored in female spermatheca (e.g., [59,60]). Female sexual receptivity only recovers when the sperm stores are exhausted [61,62,63,64], and the female likely decides the timing of remating. The mating duration, which is one of the indices of ejaculate volume, and the amount of ejaculated sperm did not change among the treatments (Figure 2 and Figure 4), suggesting that male ejaculation volume, or at least sperm volume, did not influence the mechanism of remating in *E. postfasciatus* [49,65]. In addition, the size of the spermathecae of females did not differ between the AD and SP treatments (Figure S2), indicating that it did not significantly affect the female refractory period in this weevil. These results suggest that the difference in the refractory period between AD and SP is not due to the quantitative difference in the ejaculate, or at least sperm. As the number of sperm in the male testes-seminal vesicles increases, it is considered that the number of ejaculated sperm also increases. However, the amount of ejaculate in this experiment did not change significantly between treatments (Figure 4 and Figure 8). If the spermatheca of female is hard, as in weevils, some sperm may not fit into the spermatheca if the male ejaculates excess sperm into the female. As a result, the number of sperm in the spermatheca will be less than the number of sperm ejaculated by the male.

The male uses Sfps to induce sexual refractoriness in females physiologically. Males transfer the sexual receptivity-inhibiting substances secreted by the accessory glands to females during copulation (reviewed [14,66,67,68,69,70,71,72,73,74]). Male *E. postfasciatus* inhibits female remating using Sfps from accessory gland B (AGB) [47]. It was found that the proteins in AGB differed depending on the larval diet (Figure 5A,C). One specific protein was identified in the SP treatment. In contrast, there was no qualitative difference in the proteins contained in the testes-seminal vesicle complex and AGC between SP and AD treatments. Although it has a shorter length of refractory period than the SP treatment and lacks the specific protein mentioned above, AD treatment males also inhibit female remating. This suggests that multiple proteins other than those specifically found in SP treatments are involved in the mechanism of inhibiting the female refractory period in *E. postfasciatus*. There is growing evidence that Sfps have important effects on female post-mating behavior. In *D. melanogaster,* four accessory gland proteins appear to modulate the long-term response, which is maintaining post-mating behavior and physiological changes in females [75]. In cowpea weevil, *Callosobruchus maculatus* (F.)*,* injection of a small molecular weight (<3 kDa) fraction of male Sfps results in a short-term decrease in the probability of mating, whereas the injection of a higher molecular weight (>14 kDa) fraction results in longer-term inhibition of mating [74]. In the ground beetle, *Leptocarabus procerulus* (Chaudoir), injections of either testis or accessory gland homogenates into virgin females independently decreases the probability of mating [72]. To clarify the mechanism of the refractory period in *E. postfasciatus*, it would be necessary to conduct a study in which male ejaculate is fractionated and injected to examine the response of females. Besides, further proteomic characterization, such as 2D IEF SDS-PAGE [76], will provide a basis for future studies of *E. postfasciatus* reproduction. Plastic changes in the ejaculate due to the environment during the larval stage are known, and in *D. melanogaster* the ejaculate changes depending on the rearing environment during the larval stage. Larval density environment regulated adult body size, and males that developed in low-density environments with a lager body size produced more of two key seminal proteins, sex peptide and ovulin [49]. On the other hand, in *E. postfasciatus,* male body size did not significantly influence the refractory period of females [77] and probably not the composition of the ejaculate either. To our knowledge, this is the first study to report that the larval diet qualitatively alters male Sfps.

Females could modulate the effect of Sfps by altering the sequence, levels, and expression site of the receptor [78,79]. To some extent, the physiological mechanism of the females’ response to Sfps, which affects the length of the female refractory period, is determined during the larval stage. However, the physiological aspects remain unknown. An experimental approach focusing on females is necessary to elucidate and fully understand the physiological mechanisms involved in Sfps.

### 4.3. Effects of the Amount of Sweet Potato Powder in the Artificial Diet on the Refractory Period of Females

Adjusting the amount of SP powder in the AD did not change the refractory period in females. In contrast, the amount of SP powder fed to males affected the length of refractory period of females and increasing the amount of SP powder increased the length of the refractory period (Figure 6). However, the amount of SP powder was significantly lower in the 50 g/L treatment than in the 25 g/L treatment, indicating that simply increasing the amount of SP powder did not necessarily increase the length of the refractory period. Even the 0 g/L treatment showed a refractory period of more than 10 days in both sexes. It was suggested that the length of the refractory period is determined by the physiological substance produced according to the amount of SP powder and other substances and the ratio of substances to each other in both sexes. In addition, it was suggested that the physiological responses to these substances differed between the sexes. There was no significant difference in the sperm number transferred into the spermatheca during mating between the AD and SP treatments, and as expected, this did not change when the amount of SP powder in the AD was varied (Figure 8). In the tephritid fruit fly *Anastrepha fraterculus,* adult male nutritional status affected the amount of sperm stored in females in wild flies but not in laboratory flies [30]. It was suggested that the difference between wild and laboratory females might be attributed to differences in the nutritional quality of the larval medium in which they developed. Wild flies obtained from guava are expected to be highly invasive and compete for resources. In contrast, laboratory insects are obtained from larval diets rich in carbohydrates, lipids, and proteins and have less competition for resources. Many empirical studies in insects support the hypothesis that the nutritional deficiencies in the larval stage cause delayed sexual maturation [32], reduced sperm production [24], male remating behavior, fecundity of their mates [28], and adult traits related to their reproductive success [29]. These authors suggest that insects have a nutritional threshold to reach sexual maturity, and that the nutritional level may be due to a combination of nutrients from the larval and adult diets. Teruya and Kumano [80] also suggested that the difference in the degree of sexual maturity might contribute to the difference in the experimental results of ejaculation volume in *E. postfasciatus.*

### 4.4. Application Aspects

Protein-rich diets significantly improved the ability of sterile males to inhibit female remating in *A. fraterculus* [30,31,81], *Bactrocera tryoni* (Froggatt)*, B. cucurbitae* (Coquillett), and *C. capitata* (Wiedemann) [20,22,82]. In *E. postfasciatus*, it was found that sterile males with ejaculates that strongly inhibit mated wild females from remating with wild males could be produced by changing the larval diet and could greatly contribute to increasing the efficiency of SIT. Although the sperm amount per ejaculation was not significantly different, the sperm amount stored in the testes-seminal vesicles complex was significantly different between AD and SP (Figure 3), and it increased in a dose-dependent manner for SP powder (Figure 7). Male mating behaviors and sperm production are biologically costly (e.g., [83,84,85,86,87,88]). It is presumed that there is a limit to the number of times if they have small amounts of sperm in the testes and seminal vesicles and that it will require a lot of time and energy to recover. On the other hand, if there are more sperm in the testes-seminal vesicles complex, the expected number of possible copulations will be higher. The potential number of possible mates will have a significant effect on the efficiency of SIT, and no study examined how differences in larval diet affect these factors, so further research is expected. Since our experiment used individuals that were not sterilized by irradiation, further study of ejaculates using sterilized males would be necessary to determine the actual impact on SIT.

In this study, the larval diet influenced male reproductive traits, especially those related to female remating. Male ejaculates were qualitatively different (Figure 5). Rapid evolution at the molecular level is common for reproductive proteins, including Sfps (e.g., [89,90,91,92]). Although adults of this species have well-developed hind wings, they are incapable of flight and depend on walking for movement [93]. Besides *I. batatas*, this weevil feeds on other wild *Ipomoea* plants, such as *I. pes-caprae* (L.) and *I. indica* (Burm.) [34,94,95]. It is possible that a unique evolution of the local population and field host plants occurred. Differences in ejaculate and mating behavior between local populations and field host plants in this species need to be investigated.

In SIT, control is achieved only when a sterile male copulates with a wild female, and the sperm leads to fertilization. It is important to fully understand the target pests’ reproductive behavior and traits to produce and release sterile males with strong sexual competitiveness [96]. Thus, the change of reproductive traits by the larval diet will significantly impact the effectiveness of SIT [97].

## Data Availability

On reasonable request, derived data supporting the findings of this study are available from the corresponding author.

## Supplementary Materials

The following supporting information can be downloaded at: https://www.mdpi.com/article/10.3390/insects13040389/s1, Figure S1: Photographs of male internal reproductive traits (testes, seminal vesicles, and each type of accessory gland) from SP treatment (A; upper figure) and AD treatment (B; upper figure) and female internal reproductive trait (the (a) length, (b) height, and (c) width of spermatheca) of SP treatment (A; lower figure) and AD treatment (B; lower figure) in *Euscepes postfasciatus*, Figure S2: comparison of the (A) length, (B) height, and (C) width of the spermatheca of different female treatments. Boxplots represent medians (thick lines), the first and third quartile ranges (box perimeters), minimum and maximum (whiskers), and outliers (small circles), Table S1: (A) Ingredients, quantities and (B) nutrition component in the larval artificial diets (AD) and sweet potato tuber (SP), Table S2: Estimated size of proteins (kDa) including in in the testes-seminal vesicles complex (TSVC), and accessory gland B (AGB) and accessory gland C (AGC) of *E.* *postfasciatus*. AGB No. 25, surrounded by dash lines, is an SP specific protein.
