# Supplementary material for: Effects of Larval Diet on the Male Reproductive Traits in the West Indian Sweet Potato Weevils Euscepes postfasciatus (Coleoptera: Curculionidae)
Source: Insects. 2022 Apr 14;13(4):389. doi: 10.3390/insects13040389 (PMC9031274; doi:10.3390/insects13040389)
Supplement: Supplementary file 1 [file insects-13-00389-s001.zip › insects-1595926-supplementary.pdf]

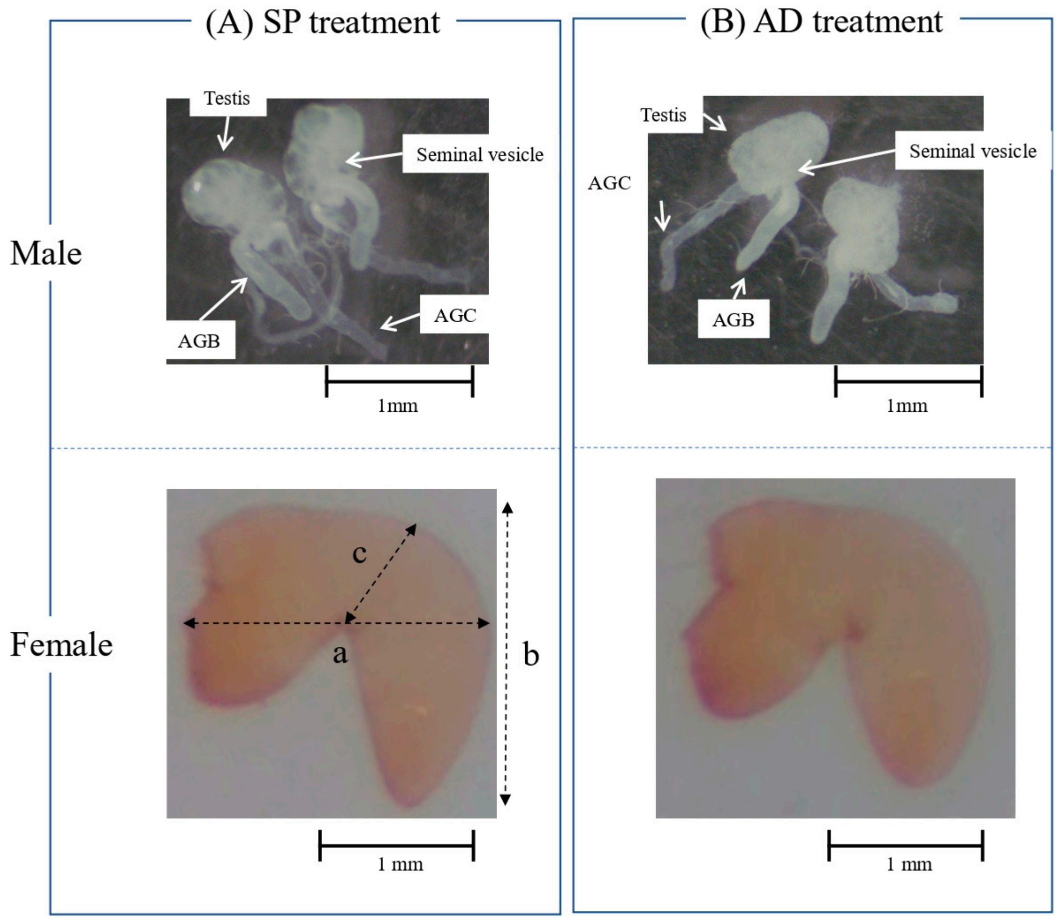


**Figure S1**. Photographs of male internal reproductive traits (testes, seminal vesicles, and each type of accessory gland) from SP treatment (A; upper figure) and AD treatment (B; upper figure) and female internal reproductive trait (the (a) length, (b) height, and (c) width of spermatheca) of SP treatment (A; lower figure) and AD treatment (B; lower figure) in *Euscepes postfasciatus*.

**Supplemental Figure 2.**

Effects of the larval diet on the size of spermatheca

Methods

To examine the difference in the size of the spermatheca, females were dissected in saline under a binocular microscope. The removed spermathecae were placed on a glass slide with a drop of distilled water (10 μL) and covered with a cover glass. Under a stereomicroscope, the spermatheca's length, height, and width were measured using imaging software (see Fig. S1, SP treatment n = 28, AD treatment n = 32). The width of the spermatheca was defined as the length of the longest point from the sharp end on the left side to the right side of the organ, as shown in the photograph. Height was defined as the length of the longest point from the lower end to the upper end of the longest part on the right side. The width was defined as the length of the line starting at the bottom hollow and extending up to the point where it is taken vertically to the top.

Statistics

We used the GLM with a Gaussian distribution and identity link function to analyze the effects of diet during the larval stage of the female, and the effect of female body size on the size of the spermatheca. The significance level for all the tests was set at 5%. All analyses were conducted using the JMP software (ver. 14.3 [56], Cary, NC).

Results

Larval diet or body size did not significantly affected the size of the spermatheca (Fig. S2 (A) length: GLM, larval diet L-R $\chi^2 = 0.05$, $P = 0.81$; female body size L-R $\chi^2 = 1.56$, $P = 0.21$; larval diet × female body size L-R $\chi^2 = 2.41$, $P = 0.12$ (B) height: larval diet L-R $\chi^2 = 0.29$, $P = 0.59$; female body size L-R $\chi^2 = 3.8$, $P = 0.051$; larval diet × female body size; L-R $\chi^2 = 3.26$, $P = 0.07$ (C) width of spermatheca: larval diet L-R $\chi^2 = 0.04$, $P = 0.83$; female body size L-R $\chi^2 = 0.34$, $P = 0.56$; larval diet

× female body size L-R $\chi^2 = 3.28$, P = 0.07).

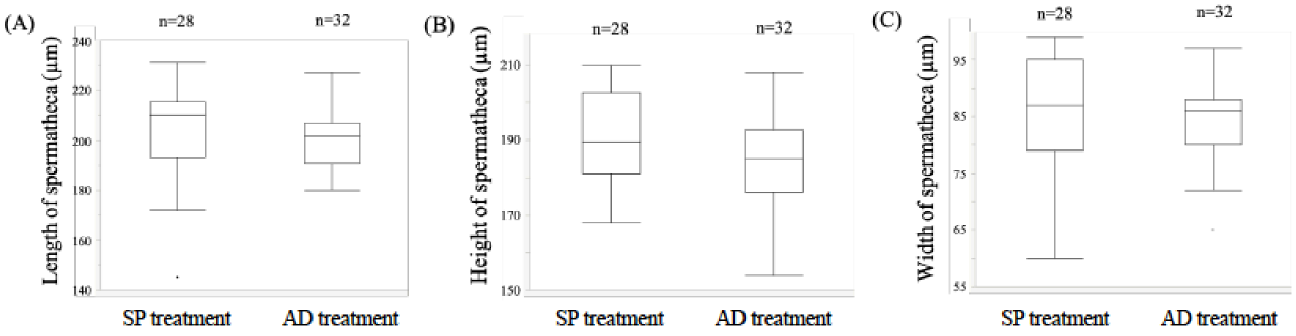


**Figure S2.** Comparison of the (A) length, (B) height, and (C) width of the spermatheca of different female treatments. Boxplots represent medians (thick lines), the first and third quartile ranges (box perimeters), minimum and maximum (whiskers), and outliers (small circles).

Table S1. (A) Ingredients, quantities and (B) nutrition component in the larval artificial diets (AD) and sweet potato tuber (SP).

† Contents of salt solution and vitamin mixture solution are referred to Okinawa Prefectural Protection Center (2018).

(A) Ingredients and quantities of AD

| Ingredients | Quantity |
| --- | --- |
| Water | 1 L |
| Agar (Ina-kanten®) | 40 g |
| Cholesterol | 800 mg |
| Potassium sorbate | 1 g |
| Methyl p hydroxybenzoate | 1 g |
| Salt mixture† | 3 g |
| Powdered cellulose (KC flock®) | 16 g |
| Powdered cellulose (KC flock®) | 40 g |
| Soy bean meal (Esusan meat®) | 24 g |
| AY-65 brewer's yeast | 10 g |
| Vitamin mixture solution† | 10 mL |
| Sweet potato powder | 50 g |
| 33% hydrochloric acid | 20 mL |

(B) Ingredients per 100g in AD and SP

| | AD | SP |
| --- | --- | --- |
| Protein | 2.3 g | 1.2 g |
| Fat | 0.2 g | 0.2 g |

| | | |
|---|---|---|
| Carbohydrate | 7.3 g | 31.9 g |

References: Ohishi et al (2018) and Standards Tables of Food Composition in Japan -2020- (Eighth Revised Edition).

**Table S2.** Estimated size of proteins (kDa) including in in the testes-seminal vesicles complex (TSVC), and accessory gland B (AGB) and accessory gland C (AGC) of *E. postfasciatus*. AGB No. 25, surrounded by dash lines, is an SP specific protein.

TSVC

| No. | Relative distance from the top | Estimated size (kDa) |
|---|---|---|
| 1 | 168 | 262.184992 |
| 2 | 188 | 224.717266 |
| 3 | 330 | 103.90208 |
| 4 | 350 | 95.8494368 |
| 5 | 374 | 87.5185044 |
| 6 | 404 | 78.733218 |
| 7 | 420 | 74.6503893 |
| 8 | 440 | 70.0379192 |
| 9 | 524 | 55.1192957 |
| 10 | 566 | 49.590162 |
| 11 | 590 | 46.8455936 |
| 12 | 638 | 42.082129 |
| 13 | 652 | 40.8482448 |
| 14 | 674 | 39.0314006 |
| 15 | 908 | 25.9400707 |
| 16 | 1040 | 21.5354597 |
| 17 | 1376 | 14.6710427 |
| 18 | 1426 | 13.9704046 |
| 19 | 1492 | 13.1301533 |

AGB

| No. | Relative distance from the top | Estimated size (kDa) |
|---|---|---|
| 1 | 166 | 266.525438 |
| 2 | 194 | 215.243816 |
| 3 | 292 | 122.875972 |
| 4 | 332 | 103.044909 |
| 5 | 358 | 92.9251401 |
| 6 | 376 | 86.8809015 |
| 7 | 410 | 77.1578623 |
| 8 | 428 | 72.744045 |
| 9 | 446 | 68.7493763 |
| 10 | 498 | 59.102442 |
| 11 | 532 | 53.9861064 |
| 12 | 586 | 47.2845459 |
| 13 | 648 | 41.1943373 |
| 14 | 680 | 38.5600097 |
| 15 | 784 | 31.7248467 |
| 16 | 878 | 27.1628836 |
| 17 | 908 | 25.9400707 |
| 18 | 972 | 23.6274256 |
| 19 | 1018 | 22.1760734 |
| 20 | 1050 | 21.2547659 |
| 21 | 1110 | 19.6955925 |
| 22 | 1144 | 18.8975183 |
| 23 | 1186 | 17.9860893 |
| 24 | 1328 | 15.4028928 |
| 25 | 1350 | 15.0598018 |
| 26 | 1378 | 14.6418575 |
| 27 | 1438 | 13.8108186 |

AGC

| No. | Relative distance from the top | Estimated size (kDa) |
|---|---|---|
| 1 | 166 | 266.525438 |
| 2 | 186 | 228.036627 |
| 3 | 284 | 127.646059 |
| 4 | 324 | 106.549066 |
| 5 | 348 | 96.6054684 |
| 6 | 368 | 89.4807251 |
| 7 | 402 | 79.2707442 |
| 8 | 420 | 74.6503893 |
| 9 | 436 | 70.9203512 |
| 10 | 488 | 60.7691663 |
| 11 | 522 | 55.409036 |
| 12 | 576 | 48.4136281 |
| 13 | 632 | 42.6308252 |
| 14 | 664 | 39.8395494 |
| 15 | 774 | 32.2881377 |
| 16 | 866 | 27.6802383 |
| 17 | 906 | 26.0186102 |
| 18 | 952 | 24.3105955 |
| 19 | 1004 | 22.6011186 |
| 20 | 1042 | 21.4788098 |
| 21 | 1098 | 19.9913004 |
| 22 | 1130 | 19.2192443 |
| 23 | 1174 | 18.2386166 |
| 24 | 1318 | 15.5633408 |
| 25 | 1370 | 14.7592047 |
| 26 | 1404 | 14.271399 |
| 27 | 1430 | 13.9168564 |
